# Designing Visual-Arts Education Programs for Transfer Effects: Development and Experimental Evaluation of (Digital) Drawing Courses in the Art Museum Designed to Promote Adolescents’ Socio-Emotional Skills

**DOI:** 10.3389/fpsyg.2020.603984

**Published:** 2021-01-18

**Authors:** Lydia Kastner, Nora Umbach, Aiste Jusyte, Sergio Cervera-Torres, Susana Ruiz Fernández, Sven Nommensen, Peter Gerjets

**Affiliations:** ^1^Leibniz-Institut für Wissensmedien, Tübingen, Germany; ^2^LEAD Graduate School & Research Network, Eberhard Karls University Tübingen, Tübingen, Germany; ^3^Faculty of Medicine, University Hospital, Eberhard Karls University Tübingen, Tübingen, Germany; ^4^FOM Hochschule für Oekonomie & Management, Essen, Germany; ^5^Herzog Anton Ulrich-Museum, Braunschweig, Germany

**Keywords:** arts education, visual arts, art museum, transfer effect, socio-emotional skills, empathy, self-concept, randomized field trials

## Abstract

An active engagement with arts in general and visual arts in particular has been hypothesized to yield beneficial effects beyond arts itself. So-called cognitive and socio-emotional “transfer” effects into other domains have been claimed. However, the empirical basis of these hopes is limited. This is partly due to a lack of experimental comparisons, theory-based designs, and objective measurements in the literature on transfer effects of arts education. Therefore, the aim of the present study was to design and experimentally investigate a theory-based visual-arts education program for adolescents aged between 12 and 19 years (*M*_age_ = 15.02, *SD*_age_ = 1.75). The program was delivered in a museum context in three sessions and was expected to yield specific and objectively measurable transfer effects. To conduct a randomized field trial, three strictly parallelized and standardized art courses were developed, all of which addressed the topic of portrait drawing. The courses mainly differed regarding their instructional focus, which was either on periods of art history, on the facial expression of emotions, or on the self-perception of a person in the context of different social roles. In the first and more “traditional” course portrait drawing was used to better understand how portraits looked like in former centuries. The two other courses were designed in a way that the artistic engagement in portrait drawing was interwoven with practicing socio-emotional skills, namely empathy and emotion recognition in one course and understanding complex self-concept structures in the other. We expected positive socio-emotional transfer effects in the two “psychological” courses. We used an animated morph task to measure emotion recognition performance and a self-concept task to measure the self-complexity of participants before and after all three courses. Results indicate that an instructional focus on drawing the facial expressions of emotions yields specific improvements in emotion recognition, whereas drawing persons in different social roles yields a higher level of self-complexity in the self-concept task. In contrast, no significant effects on socio-emotional skills were found in the course focussing on art history. Therefore, our study provides causal evidence that visual-arts programs situated in an art-museum context can advance socio-emotional skills, when designed properly.

## Introduction

### Cognitive and Socio-Emotional Transfer Effects of Visual-Arts Education

It has been hypothesized for decades that an active engagement with the arts in general and the visual arts in particular might yield a plethora of beneficial effects, within and even beyond the arts itself (Baker, 2012; Catterall et al., 2012; Bowen et al., 2013; Winner et al., 2013b). Therefore, arts education in schools and museums or other institutions is not only expected to promote so-called “*primary effects*” of arts education in terms of cultural participation or the development of receptive and productive competencies in various aesthetic and artistic forms of expression (Keuchel, 2019). Rather, beyond these intrinsic values of art for art’s sake, engagement with the arts is also seen as a potential means to achieve broader positive “side effects” that are usually labeled as “*transfer effects*” (Knigge, 2013). These hypothesized transfer effects comprise cognitive side effects regarding general academic achievement and intelligence development (Bastian et al., 2000) but also more specific abilities such as problem-solving, critical thinking, memory, or spatial and geometrical thinking (Winner et al., 2013b). Moreover, beyond these cognitive aspects expected transfer effects also cover creativity outcomes, motivational benefits, or even improvements in socio-emotional skills (Winner et al., 2013b; Watson et al., 2019). Based on these expectations, arts education has been seen by many as a valuable contribution to the development of several overarching and domain-general skills or competencies usually referred to as “21st-century skills” (Dede, 2010). This argument was used not only to stress the relevance of school subjects such as visual arts or music, but also to underline the importance of extracurricular cultural institutions such as art museums (Aspin, 2000; Schönau, 2012; Savva, 2013).

However, the empirical basis toward broad beneficial effects caused by an active engagement with art in general and visual arts in particular is severely limited. For instance, it has been argued in a meta-study by Winner et al. (2013b) that substantial empirical research with regard to transfer effects of an engagement with the visual arts (and many other art forms) is largely missing. According to Winner et al. (2013b), studies on this topic are not only quite rare and ambiguous in their results but also rather unsatisfactory in terms of their methodological and theoretical validity. Most importantly, only a small proportion of the available studies are based on randomized experimental designs, which would be required for a causal attribution of transfer effects to visual arts engagement. The authors claim that reliable causal evidence for positive cognitive side effects of visual-arts education has been found only in one case, namely with regard to *observational skills*. It could be demonstrated experimentally that inspecting artwork also improves the quality of observing scientific images within a medical (Dolev et al., 2001) or a biological context (Tishman et al., 1999).

All other cases in the domain of cognitive “side effects” of visual-arts education seem to be less clear. For instance, there is correlational evidence that visual-arts education is related to *geometric and spatial reasoning skills* (Spelke, 2008; Walker et al., 2011). Moreover, a causal link between visual arts engagement and geometric or spatial reasoning skills is theoretically quite plausible. However, a meta-analysis of 30 quasi-experimental studies on this relation yielded no significant effect (Haanstra, 1996). Moreover, the respective longitudinal evidence is also weak since no relation between the development of drawing and of geometric reasoning skills over time could be demonstrated (Winner et al., 2013a). Thus, the correlational evidence found might not represent a causal link but rather a self-selection effect in which students with better spatial reasoning skills might more often decide to engage in visual-arts education programs.

Other examples of disappointed expectations with regard to hypothesized cognitive “side effects” of visual-arts education are findings showing that engagement in visual arts (in contrast to other art forms such as theatre or dance) did not stimulate *creativity* or *problem-solving skills* (Catterall and Peppler, 2007). Winner et al. (2013b) interpret these findings as showing that “there is no reason to think that arts education will make children more creative unless the arts are taught in a way that really pushes children to explore and invent” (p. 196). In sum, the evidence for transfer effects of visual-arts engagement on generic cognitive skills is limited, which might partially go back to the issue that this engagement is often not tailored toward specific and intended transfer effects.

When it comes to hypothesized transfer effects besides the cognitive domain, the empirical situation seems to be difficult as well. There is little reliable experimental evidence for socio-emotional transfer effects of visual-arts education. Instead, there is mostly correlational or quasi-experimental evidence prone to self-selection effects. This applies to the relation between visual arts engagement and a better development of the self-concept (Catterall and Peppler, 2007), a better regulation of emotions (Goldstein et al., 2013) or an increase in empathy with other persons (Goldstein and Winner, 2012). Moreover, the few experimental studies on socio-emotional transfer effects of visual-arts education usually fail to demonstrate the expected effects. For example, in the experimental studies by Greene et al. (2014, 2015) matched school classes were randomly assigned to either participate or not participate in an art-museum field trip. While some effects were detected regarding cognitive and motivational outcomes, no effects of this experimental manipulation were found on empathy measures or social perspective taking. In a follow-up longitudinal experimental study on multiple arts-based field trips by Erickson et al. (2020), control students were compared to students who attended three different art field trips, visiting an art museum, a live theater performance and a symphony. Again, effects were found toward cognitive and motivational outcomes, but no effects were found on empathy measures or social perspective taking.

According to Winner et al. (2013b) this lack of socio-emotional outcomes of standard visual-arts education is theoretically not very surprising. Visual-arts education usually does not involve imagining oneself in unknown roles and situations, expressing strong emotions, or stepping in the shoes of other persons (other than, for instance, theater education for which socio-emotional effects have been demonstrated empirically). Therefore, one might argue that there is no reason to expect that visual-arts education will make children *per se* more competent with regard to socio-emotional skills unless the arts are taught in a way that really pushes children to explore their own self-concept, to express strong emotions or to empathize with other persons’ perspectives and feelings. When these aspects are not a pivotal part of a visual-art education program, this program might just not provide sufficient opportunities for improving these socio-emotional skills. In other words, an artistic engagement that does not teach, practice, presuppose or reflect a particular skill or competence will probably make no substantial contributions to its development and improvement.

Based on similar assumptions, Hetland et al. (2013) started to systematically observe which activities might constitute pivotal ingredients of high quality visual-arts classrooms. From these observations they derived a list of expectations regarding potential transfer effects of visual-arts education. As a result, they came up with eight cognitive and motivational skills that seemed to be fundamental to any artistic engagement in the field of visual arts and to any program for visual-arts education. They described these skills as “studio habits of mind” and conceptualized them as crucial mental processes in creating visual artworks. Taking into account the crucial role of these skills for visual-arts education, the authors considered them to be the theoretically most plausible candidates for general transfer effects that might be improved by an active engagement with visual arts. These skills are (1) developing crafts, (2) engaging and persevering, (3) envisioning something, (4) expressing oneself, (5) observing closely, (6) reflecting, (7) exploring and (8) understanding artistic worlds. It is noticeable, though, that socio-emotional skills such as the ones mentioned above (self-concept development, emotion regulation, empathy) are not included in this list of studio habits of mind. These skills seem not to be at the core of what is usually being taught in visual-arts classrooms and therefore might have a smaller chance of being practiced during art making than other skills that are considered studio habits of mind. This could explain why corresponding transfer effects on socio-emotional skills have not yet been demonstrated for “standard” visual-arts programs.

A seemingly lower importance of socio-emotional skills for visual arts programs, however, does not rule out the possibility to develop specific programs with an additional socio-emotional focus. For instance, visual-arts engagements could be tailored in such a way that they strongly stimulate participants to image themselves in unknown roles and situations, to express strong emotions, or to step into the shoes of other persons just as it has been demonstrated for other forms of art engagement that yield socio-emotional transfer effects such as theater education (Winner et al., 2013b). Under these circumstances, it might even be possible that conveying socio-emotional skills in a visual-arts context is particularly effective due to the studio habits of mind involved. A specific artistic assignment focusing on socio-emotional contents could be more intense when studio habits of mind, such as precise observation, engagement, imagination, reflection, etc. are involved compared to assignments using other art forms like music or dance without them. Consequently, synergies between the generic characteristics of visual-arts engagement in the sense of studio habits of mind and the more specific contents of an artistic assignment designed to address socio-emotional skills might occur. This could result in supporting the understanding and skill development in this area thereby evoking socio-emotional transfer effects. Similar synergistic arguments have been raised for drawing as a generative learning tool in other domains (Mayer and Fiorella, 2014; Fiorella and Mayer, 2016). Therefore, the idea pursued in this paper is to use the habits of mind, which are specific for both the understanding and making of art and to combine them with artistic contents of socio-emotional nature. We assume that this allows for the first time to establish an experimental benefit for visual-arts programs by the specific habits of mind, in which they can evoke socio-emotional transfer effects. The prospects of this idea will be investigated in the study reported in this paper.

### Methodological Boundary Conditions for Testing Transfer Effects in Art Education

For testing socio-emotional transfer effects of specifically designed visual-arts programs, three important methodological boundary conditions can be derived from shortcomings of the existing literature on transfer effects in art education. These boundary conditions are related to experimental comparisons, theory-based designs and objective measurements.

#### Experimental Comparisons

First, most studies in the field of transfer effects investigate art-education programs that have already been implemented and attended by (self-selected) participants. Usually, either (1) correlative relationships between outcomes and participation (or not) in a program are analyzed or (2) differences in outcomes are examined in form of quasi-experimental comparisons of two programs. However, due to self-selection, persons participating in either program may differ in various other characteristics than group membership. Therefore, no causal conclusions can be derived from group membership alone, as any of these other (confounding) differences might be responsible for differences in outcomes. Moreover, when comparing quasi-experimentally two programs that differ not only with regard to a variable of theoretical interest (e.g., artistic approach), any other difference between these programs in terms of structures, contents or instructors might also be responsible for potential differential effects. Accordingly, to derive causal conclusions on transfer effects of visual-art education programs, randomized experimental comparisons between specific programs are necessary, that should only differ with regard to characteristics of theoretical interest. Therefore, we implemented a randomized field trial with a randomization of school classes at the student level. Students visited an art museum to participate in one of two simultaneously conducted visual-arts courses. The courses were designed for maximum equivalence except for characteristics of theoretical interest. This approach avoids the detrimental confounds and self-selection effects mentioned above. Since students are obliged to join their class, also differential attrition effects (i.e., dropouts in experimental groups) over the course of the program can be controlled. From an experimental view, studying courses in an art museum (instead of a school context) has the advantage that it allows the implementation of highly controlled courses which are run by identical persons in identical rooms for all classes and across all experimental groups. Thus, it avoids important confounders regarding the courses, the self-selection of participants, and the characteristics of particular school classes (e.g., school type or school district). Since this setting requires a between-subject design (i.e., each student participates only in one of two parallel courses), sufficient group sizes are important to ensure substantial statistical power to identify small effects that may be expectable from the literature on transfer effects of visual-arts education.

#### Theory-Based Designs

A second methodological limitation of the existing literature on transfer effects of visual-arts education might be more theoretical. In particular, the fact that this literature has generated only little reliable evidence to support transfer effects might not alone go back to the abovementioned flaws of study designs. Instead, there may also be more fundamental flaws regarding the theoretical reasoning pertaining to the design of visual-arts programs and the choices of dependent measures in this research. From a psychological perspective, it would be considered important to base the design of visual-art programs aiming at specific transfer effects on a deep theoretical understanding of the targeted core constructs. If, for example, transfer effects on target constructs such as empathy are intended, the underlying theories of psychological mechanisms and preconditions should be well understood by the program designers. Otherwise, a lack of theoretical understanding might prevent them from designing effective programs. Moreover, also the development and/or selection of measurement instruments that are suitable for demonstrating transfer effects on target core constructs might require theoretical in-depth knowledge. Thus, if an art program is intended, for instance, as an intervention to support participants’ empathy or to improve the complexity of their own self-concept (both of which were aims in our study), the design of the respective programs and measures needs to be based on three theoretical pillars: (1) Sound psychological models and measures of empathy and self-complexity, (2) theoretical insights on how these skills can be conveyed and trained, and (3) theoretical knowledge about potentially important personal preconditions that might moderate the effectiveness of interventions. If art programs which aim at the empirical demonstration of specific transfer effects are not based on these important theoretical pillars they may easily fail to achieve these goals. Accordingly, in the current study we considered it not to be sufficient to start with a rather superficial conception of socio-emotional skills, such as empathy or self-complexity, when designing a visual-arts program that is supposed to support the development of these skills. Similarly, we assumed that the development of measurement instruments that are suitable to validly assess skill levels and skill developments with regard to specific constructs would require precise theoretical models of the target skills adressed.

#### Objective Measurements

A third methodological limitation for testing socio-emotional transfer effects of specifically designed visual-arts programs might be to strive for more objective measurements. Whereas cognitive transfer effects of visual-arts education (for instance, effects on spatial and geometric reasoning, problem solving, or observational skills) are usually assessed by means of objective performance measures, socio-emotional transfer effects are usually assessed more subjectively. For instance, questionnaire data or self-reports rather than performance measures are typically used to assess constructs such as self-concept or empathy. Relying on subjective data, however, might be one of the reasons why existing studies have not yielded strong evidence for socio-emotional transfer effects of visual-arts education, yet. For instance, questionnaire data might not be as sensitive as necessary to track small changes with regard to specific aspects of socio-emotional skills resulting from a visual-arts program. Additionally, it is well known that the correlation between objective skills and subjective reports on them might be rather weak (Dang et al., 2020). Thus, if assessements of outcomes could be based on objectively measurable aspects of socio-emotional skills, there might be a better chance to track the skill development trajectory during a visual-arts program. To be more concrete, if a visual-arts program that is intended to support participants’ empathy or to improve the complexity of their own self-concept is to be evaluated, it might not be the best approach to ask them before and after the program about their deemed ability in perceiving and understanding the emotions of others or in different roles and situations. Nevertheless, this is basically the approach taken by many studies in the field (e.g., Catterall and Peppler, 2007; Goldstein et al., 2013). A better approach would possibly be to obtain their ability to perceive and understand the emotions of others in an objective test or to assess the complexity of their self-concept based on concrete self-descriptions in the context of different roles and situation. These performance measures, as opposed to self-reports, would better allow to directly assess how good participants’ emotion perceptions are and how differentiated their self-representation is. Therefore, objective measures might not only be more sensitive to track skill developments but also more valid in measuring the constructs of interest than measures asking for the subjective impressions of participants. Accordingly, we deployed objective measures to evaluate two theory-based art programs, designed to either support participants’ skills of perceiving emotional states in others based on their facial expressions (which is an important subcomponent of empathy; cf. Besel and Yuille, 2010), or their ability to perceive themselves in different roles and situations (which is at the core of self-complexity; Linville, 1985; Woolfolk et al., 2004).

### Target Constructs for Transfer Effects: Empathy and Self-Concept Development

In the literature it has been hypothesized, but not yet demonstrated empirically, that visual-art education might have beneficial transfer effects on the development of the self-concept (Catterall and Peppler, 2007), the regulation of emotions (Goldstein et al., 2013), or the ability to experience empathy with other people (Goldstein and Winner, 2012). Based on our previous theoretical considerations, it seems indeed plausible that visual-arts programs could be designed in a way to comprise artistic assignments, which motivate children to empathize with other persons’ perspectives and feelings, or to elaborate on a multi-faceted self-concept. It is less clear, from our perspective, how a visual-arts engagement might be deployed to stimulate reappraisal processes in order to change one’s own strong negative emotions. Based on these intuitions, we focused on constructs related to empathy and self-concept development in our study.

#### Empathy

Empathy, conceived in a very broad way, can be understood as the general ability to perceive the situations, thoughts and feelings of other individuals correctly. Empathy can be seen as a main factor influencing individuals’ well-being and relationship quality but also as a requirement for good communication skills and understanding of other people’s perspectives and emotions (Ioannidou and Konstantikaki, 2008). It has been argued that engaging in empathy tasks (i.e., trying to empathize with another person) not only requires but also promotes empathy in the sense that practicing empathy reinforces it, which might be a good precondition for designing a visual-arts program that is intended to support empathic skills (Winner, 2019). Two levels of empathy have been distinguished, namely cognitive and emotional empathy. Cognitive empathy refers to the cognitive ability to recognize the mental and emotional situation of another person, i.e., to correctly assess what another person feels or thinks. Emotional or affective empathy (that builds on cognitive empathy) is characterized as the ability of a person to empathize with another person’s emotional experiences (e.g., feeling pity or compassion, but also the happiness of another person).

It has to be noted that previous studies hypothesizing (but not demonstrating empirically, yet) that visual-art education might have beneficial effects on empathy (Goldstein and Winner, 2012) used standard empathy questionnaires, which assess the extent to which a child shows an emotional reaction to another’s emotional situation, as dependent measures. These measurements, however, do not distinguish between the abilities to perceive and to share others’ emotions. From a theoretical perspective, it is more plausible that suitable visual-arts education programs, focusing on an artistic engagement with emotional expressions, will mainly support students’ cognitive empathy in the first step. This ability to correctly recognize other peoples’ emotions is a key element of competent social behavior in many everyday situations (Ekman and Friesen, 2003) and develops crucially during adolescence (Olderbak et al., 2018). Moreover, it is a main precondition for (but not the same as) sharing other peoples’ emotions (cf. Besel and Yuille, 2010). We assume that studio habits of mind such as expressing (one’s own or others’ emotions) in authentic drawings, observing (social and emotional behaviors) and reflecting on them might be practices that are closely related to cognitive empathy. They require but also promote and thereby reinforce this skill. We hypothesize that particularly cognitive empathy might benefit from visual-arts engagements which deploy studio habits of mind to address issues of emotion expression. To test this idea more directly, we decided to measure emotion recognition performance as an objective outcome measure of a visual-arts education program instead of using empathy questionnaires comprising cognitive and affective aspects of the construct.

Among other things, bodily and particularly facial expressions of emotions are used as central cues for emotion recognition. It has be shown that difficulties in recognizing emotions on the basis of facial expressions are related to clinical disorders of social behavior such as autism (Harms et al., 2010). However, it has also been shown that the emotional recognition performance can be improved in a targeted manner by means of instructions, for example by directing attention to the parts of the face that are relevant for emotion recognition. This has, for instance, been demonstrated in clinical studies on autism (Harms et al., 2010) or schizophrenia (Silver et al., 2004). For non-clinical populations it has also been demonstrated that an intensive drawing engagement with portraits leads to a more analytical and less holistic—and thereby improved—processing of faces (Zhou et al., 2012). Balas and Sinha (2008) found similar results. They argued that people without artistic portrait drawing experience seem to be more prone than experienced portrait drawers to misrepresent important facial features. These findings provide first evidence for the assumption that applying studio habits of mind such as closely observing, envisioning, or reflecting may indeed lead to better, more detailed and less distorted representations of facial features. In the design of one of our visual-arts education programs, which was intended to support adolescents’ cognitive empathy, we have taken up this assumption about the beneficial role of the studio habits of mind for improving the processing and detailed representation of faces. Here, the idea was to promote cognitive empathy as a basic precondition of empathic behavior by means of an artistic engagement with portrait drawings focusing on emotion expression.

#### Self-Concept Development

The self-concept can be understood as a collection of all potentially accessible beliefs that a person holds about him- or herself (Aronson et al., 2008). The self-concept has been described as a multidimensional construct consisting of many different facets that develop over time. In previous studies hypothesizing that visual-art education might have beneficial effects on the development of the self-concept (Catterall and Peppler, 2007) the focus was mainly on ability beliefs, self-efficacy beliefs and beliefs about the causes of personal success and failure. Thus, the questionnaire data obtained in this research may have mainly tapped into very general competence beliefs. One might ask, however, why the engagement in visual-arts education should be expected to improve one’s general competence beliefs. If, at all, one might expect improvements with regard to more specific and art-related competence beliefs addressing abilities to envision, create, design and finally even produce artistic results. It remains unclear, however, why improvements with regard to these beliefs (that have not been measured) should generalize to other domains.

An alternative and from our theoretical perspective more plausible artistic approach to self-concept development would be to focus on the complexity of the self-concept: First, it can be assumed that an artistic engagement in the visual arts might be useful to explore and elaborate a multi-facetted view of other persons’ self-perception in their diverse social roles and situations, for instance, by means of drawing portraits of persons in diverse roles and situations. This engagement might be conceived as a high-level empathy task requiring not only the basic perception of isolated feelings of others but also a deeper imagination of their self-perceptions in different and potentially conflicting roles and situations. Such a task might, in a second step, stimulate a more complex and differentiated scheme for person perception that might, eventually, also transfer to the perception of one’s own person in different roles and situations. The core idea would be—just as the perception of one’s own emotions might provide a basis for the perception of the emotions of others—that a more differentiated approach to the exploration of other persons’ self-concept might eventually apply when considering the characteristics of one’s own person in different roles and situations. Therefore, we would argue that a visual-arts education program, even if it does not improve one’s general competence beliefs, still might be suitable to support self-concept development in terms of a better differentiation of the self-concept.

To work out this idea in greater detail, we can refer to McConnell’s (2011) Multi-Self-Aspect Framework. There the self-concept consists of a network of self-aspects such as social roles and the associated attributes of one’s own person, which can vary in their accessibility. The differentiation of the self-concept can be described in this model as a nuanced access to characteristics of one’s own person in the context of various social roles. For example, in the school-context, the role of a student with the corresponding characteristics of one’s own person might be more easily accessible than the role of a daughter or son that might be associated with a different set of personal characteristics. Possessing a differentiated self-concept along this line of thought has also been described as self-complexity (Linville, 1985, 1987; Woolfolk et al., 2004). Being able to perceive many facets of one’s self in a differentiated way has also been claimed to be a fundamental process for the maintenance of general well-being and thus for success in various areas of life (Rafaeli-Mor and Steinberg, 2002). To possess a complex self-concept might prevent a person facing a failure in one area of life, e.g., a bad mark in mathematics, to also draw negative conclusions with regard to other areas of life, eventually leading to a general negative self-evaluation as a person. Hence, a complex self-concept has also been postulated as a protection against clinical disorders such as depressive reactions (Linville, 1987; Rafaeli-Mor and Brown, 1997). We have taken up these theoretical considerations and empirical findings in the design of our second visual-arts education programs, which was intended to support adolescents’ self-concept development. Here, the idea was to promote self-complexity by using an artistic engagement with portrait drawings that requires imagining how another person’s self-perception might look like in different and conflicting roles and situations. The students should focus on the different characteristics of their personality in different roles and situations to develop a complex self-representation which might eventually also transfer to one’s own self-perception.

### The Present Study

In the present study we investigate, in an art-museum context at the Herzog Anton Ulrich-Museum (HAUM) in Braunschweig, Germany, whether the engagement with a tailor-made visual-arts program can yield positive transfer effects on specific socio-emotional skills. Furthermore, we explore how these effects might depend on the design of the program and on the personal preconditions of participants. We focus on empathy (in the sense of emotion perception) and on self-concept development (in the sense of self-complexity) as socio-emotional target constructs for transfer effects. We also designed two visual-arts programs, each intended to contribute to the development of one of these target constructs. In designing the programs, we used not only the museum pedagogical course concepts deployed in traditional art courses at the HAUM but also approaches and methods from instructional psychology on how to teach, practice, and reflect particular skills or competences in order to contribute to their development and improvement. Most importantly, we also tried to embed as many studio habits of mind as possible in the visual-arts education programs due to the abovementioned theoretical considerations on their amplifying potential when dealing with content domains in the mode of artistic drawing. With regard to the overall content domain of the course programs (portrait drawing), additional expertise from art history and art pedagogy were deployed to guide many design decisions.

For experimental comparison, three standardized art courses were developed, which mainly differed in their instructional focus but were otherwise held strictly parallel. All courses conveyed knowledge on drawing portraits and comprised artistic portrait-drawing experiences related to artworks in the museum as well as to selfies taken by the participants. Each course lasted 9 h and took place at the HAUM over a period of 3 weeks (one session per week). All participants of the courses were provided with a tablet computer and a drawing app specifically designed for the courses to ensure the availability of support tools allowing to explore novel digital forms of artistic expression.

We introduced a different instructional focus for each of the three courses as experimental variation. One course explicitly addressed the intended socio-emotional transfer effects of correctly perceiving subtle emotional expressions in faces as a basic skill involved in empathy. In this “emotion course,” portrait drawing was used to provide experiences with the perception and interpretation of one’s own and others’ emotions based on facial expressions. All six basic emotions were addressed in the course with a focus on the two socially most important basic emotions anger and fear, which were targeted in the drawing assignments. We expected that participants’ abilities to interpret subtle emotional expressions in faces (in particular with regard to the emotions anger and fear) would improve due to course participation. A second course focused on a different target for transfer effects of visual-arts education, namely self-concept complexity. In this “self-concept course,” portrait drawing was used to explore artistically in detail how a person might experience themself differently in the context of diverse social roles the person is involved in. We expected that participants’ abilities to provide a differentiated representation of their own self-concept structure with regard to how they experience themselves differently in diverse social roles (i.e., self-complexity) would improve due to course participation. A third and more conventional course was used as a baseline control group. It focused on characteristics of portrait drawings in different periods of art history, e.g., hair accessories or clothing styles in the Baroque vs. Rococo period. In this “epoch course,” no psychological contents or constructs were addressed, and portrait drawing was mainly used to explore the artistic expression in portraits of different periods of art history.

The data collection for the study reported in this paper took place as a part of a larger study comprising two phases of data collection with two cohorts of students. In the first data collection phase, the emotion course and the epoch course were offered simultaneously to individual school classes at the HAUM, which allowed for randomization at the student level. In the second data collection phase, the self-concept course and the epoch course were offered simultaneously to a different set of school classes with randomization at the student level. The participating school classes were split, and students were randomly assigned to one of the two courses in each data collection. To ensure randomization and to avoid self-selection effects, the students were not allowed to switch their courses. Students participated in only one course of the two courses offered in each data collection phases. In both data collection phases, we used a pre- and posttest-design in which the students had to complete emotion-recognition and self-concept tasks before and after participating in the course program. These are the two tasks that will be examined more closely in the present study.

In addition to these measures, process data and questionnaire data on personal characteristics were obtained as part of the larger study for later analyses on relations between drawing processes and personal characteristics. At the process data level, the students’ drawing data (time pencil on paper, number of (erase) strokes, pressure (*M*, *SD*), altitude (*M, SD*) number of used tools/colors, number of zoom-in and fade-out interactions) were recorded as they completed the drawing tasks. With regard to personal characteristics, we additionally measured the students’ empathy skills by means of questionnaires (*Toronto Empathy Questionnaire*, TEQ, Spreng et al., 2009; *Toronto Alexithymia Questionnaire*, TAS, Bagby et al., 1994a,b; *Questionnaire on Perception of Others/Fragebogen zur Wahrnehmung der Emotionen anderer*, FWEA, Lischetzke, 1999) and the *Multifaceted Empathy Test* (Foell et al., 2018). Moreover, *personality traits* (HEXACO-PI-R, Lee and Ashton, 2004; Moshagen et al., 2014), verbal intelligence using a *vocabulary test* (WST, Schmidt and Metzler, 1992), and clinical characteristics measured with questionnaires such as the *Inventory of Callous Unemotional Traits* (ICU, Essau et al., 2006; Ciucci et al., 2013) and the *Autism Questionnaire* (AQ, Baron-Cohen et al., 2009) were assessed. We also tested students’ knowledge about emotions, epochs and the self-concept. However, the knowledge test about the self-concept was only included in the second data collection phase so that knowledge-test data does not exist for all three courses. Therefore, we have not included knowledge-test data in the current study. From the abovementioned set of additional process data and data on personal characteristics, in the current study only the TEQ data on empathy skills will be included as a potential moderator variable in the statistical analyses. The following hypotheses will be tested in the current study:

Hypothesis 1 (*Specificity Hypothesis*): Programs designed specifically for one particular socio-emotional transfer effect (e.g., on the recognition of other persons’ emotions) are hypothesized to improve this skill but not necessarily closely related skills (e.g., the complexity of one’s self-concept).

In both courses designed for transfer effects in this study, participants engage in portrait drawings in the context of imagining the situation of another person and empathizing with this person. Nevertheless, we do expect differential effects due to the focus on different contents of the empathizing during portrait drawing, namely the emotion recognition vs. the self-perception in diverse social roles. Due to these differences, the courses provided differential opportunities to engage with specific socio-emotional skills that are expected to influence the course outcomes. Accordingly, we would not expect substantial effects of the emotion course on self-complexity or of the self-concept course on the ability to interpret subtle emotional expressions. Based on this reasoning, we can also derive a second (null-)hypothesis with regard to the epoch course, in which students were also drawing portraits and studying faces of others and themselves intensively, but without an instructional focus on socio-emotional contents:

Hypothesis 2 (*Instructional Focus Hypothesis*): We do not expect transfer effects on socio-emotional skills for the epoch course used as baseline control group due to a lack of instructional focus on these skills and a corresponding lack of opportunities to engage with these skills.

Thirdly, based on theoretical considerations put forward in section “Empathy” we assume that entry levels of socio-emotional skills might moderate the effectiveness of the two visual-art education programs designed for socio-emotional transfer effects. In both courses, students are strongly encouraged to empathize with the person they focus on in their portrait drawings. In particular, the artistic drawing assignments in both “psychological” courses were designed as opportunities to imagine other persons’ perspectives and feelings. For instance, students were asked in the emotion course to interpret the emotional expression in an artwork and to render the same expression even stronger in their own drawing. Or they were asked in self-concept course to imagine how a person in an artwork perceives herself in a particular role and situation and to express this self-perception by means of drawing. We assume that engaging in this type of empathy tasks does not only require but also promotes empathy in the sense that practicing empathy improves empathy. Therefore, we expected that students with lower empathic skills may benefit more from the course programs due to two reasons: (1) Lower-empathy students might have experienced less opportunities so far to engage in specific activities focusing on empathy meaning that the opportunities provided by the “psychological” courses might play a bigger role for them than for participants with higher empathy levels. (2) Lower-empathy students might have more room for improvement left with regard to relevant skills and thus may yield a steeper learning curve. Hence, we assumed that a generic measure of how well participants already perform in different types of empathy-relevant situations (as measured by the TEQ as a standard compound questionnaire measure of empathy) might provide a potential moderator of course effects:

Hypothesis 3 (*Entry-level Hypothesis*): Participants with lower empathetic skills may particularly benefit from courses addressing socio emotional skills such as emotion recognition or self-complexity.

## Materials and Methods

### Participants

A total of 294 adolescents of grades 7–12 of all school types located in the city of Braunschweig (central Germany) participated in the present study. The study was approved by the local ethics committee and by the regional council of Braunschweig (Germany). Participating classes were recruited by the head of the museum pedagogy of the HAUM in Braunschweig. The final sample was composed of 137 girls, 112 boys and 45 adolescents without gender information aged between 12 and 19 years (*M*_age_ = 15.02, *SD*_age_ = 1.75). Within each class, the adolescents were randomly assigned to one of two parallel courses. We conducted two phases of data collection with two cohorts of students (for more information, cf. section “The Present Study”). In a first data collection phase, the emotion course was offered in parallel to the epoch course, while in a second data collection phase, the self-concept course was offered in parallel to the epoch course. The baseline control group was oversampled by offering the epoch course in both collection phases to increase the statistical power of subsequent analyses (in particular with regard to the (null-)hypothesis 2 that there are no transfer effects in the baseline course) and, thus, to obtain more conclusive results.

### Design, Procedure, and Course Program

To study differential effects of the courses as well as changes over time on socio-emotional skills, pre- and post-measurements were collected before and after the experimental intervention. Transfer effects were measured with two tasks (for details cf. section “Stimuli and Apparatus”): (1) an *animated morph task* to measure individuals emotion recognition performance (Schönenberg et al., 2013), and (2) a *self-concept task* based on a graphical form to measure the complexity of their self-concept (Aron et al., 1992). Before the study was conducted and the course started, all adolescents, as well as their parents, had to read and sign a form of consent. Adolescents who did not receive permission from their parents to participate in the study still had the opportunity to take part in the course program without data collection. The courses were conducted by two museum pedagogues of the HAUM who had previously been trained by means of a course manual (Kastner et al., 2020a). In the course manual, all three course sessions of the three different courses were specified in detail. The museum pedagogues took turns in conducting the different courses to avoid confounds due to course-teacher effects. They were not aware of the hypotheses investigated in the study. Moreover, they did not collect the pre- and posttest measures. These measures were obtained by the experimenter in the class setting while the museum pedagogues were sitting in front of the class. The course programs of the three different courses are described in detail in the following paragraphs to make clear, how the different course contents have been interwoven with the eight studio habits of mind, namely (1) developing crafts, (2) engaging and persevering, (3) envisioning something, (4) expressing oneself, (5) observing closely, (6) reflecting, (7) exploring and (8) understanding artistic worlds.

In a first course session, (1) declarative knowledge with regard to the respective course contents was conveyed, (2) accurate observation was trained, and (3) the course contents were illustrated and exemplified with artworks from the museum’s collection (analog to the *Demonstration-Phase* described by Hetland et al., 2013; Hogan et al., 2018). In the art exhibition, adolescents’ understanding of artistic worlds could be stimulated and they could deepen their acquired knowledge by exploring and discussing artworks that fit to their respective course theme. Here, the focus was on studio habits such as observation and reflection. At the end of the first course session, in each course two worksheets were handed out as homework, which required students to rehearse course contents and to apply these contents to another piece of art.

To be more concrete, in the emotion course, students first learned about characteristic features observable in facial expressions of the six individual basic emotions *happiness, anger, sadness, fear, surprise*, and *disgust* (e.g., when anger is expressed, the eyebrows tighten, and the gaze is rigidly focused straight ahead). Moreover, they reflected, on why it is important to be able to recognize non-verbal facial expressions of emotions quickly and reliably in social situations. Afterward, the adolescents had the opportunity to identify basic emotions in different paintings in the exhibition. For instance, they were asked to explore the painting ‘‘Cain slays Abel’’^1^ by Gioacchino Assereto (Genoa, 1600–1644). The painting should be closely observed, reflected critically, and discussed in detail with regard to which emotional expressions are displayed exactly.

In the self-concept course, students first learned about the structure, function and development of the human self-concept and about how to integrate within a coherent representation of the self-potentially conflicting personal characteristics associated to different competence-oriented or relationship-oriented roles. Moreover, students were asked to reflect on their own personal characteristics in different situations and on important social roles they take in their lives. We used the painting ‘‘Family Picture’’^2^ of Cornelis de Vos (1618–1619) from the HAUM exhibition to get students engaged with the topic of self-complexity in an authentic art context. Students were asked to identify themselves with a girl sitting in front of a piano in the center of the painting, looking toward the observer while being surrounded by her family. Students were instructed to detect, reflect and discuss the different roles the girl might potentially take in her daily life, such as *pianist* (because she is sitting in front of the piano), *daughter* (because it is a family portrait and her parents are also depicted), *sister* (because the whole family is depicted in this portrait, or *fiancée* (because she is wearing a ring) who is supposed to marry somebody she might love or not. The focus of this exploration was to consider how different she might perceive herself in the different roles.

In the epoch course, students were first given a general art historic introduction to portrait drawings from different centuries and learned about the stylistic characteristics of different art epochs. Subsequently, students focused more specifically on the differences between portraits from the Baroque and Rococo periods and had the opportunity to study two typical examples for each of the two periods in the HAUM exhibition. For the Baroque period we selected two portraits painted by Hyacinthe Rigaud, namely the ‘‘Portrait of Duke Anton Ulrich of Braunschweig-Wolfenbüttel’’^3^ (Paris, 1680--1693) and the ‘‘Portrait of Elisabeth Charlotte d’Orléans, née Countess Palatinate near Rhine’’^4^ (Paris, 1716). For the Rococo period, students explored the ‘‘Portrait of Maria Antonia Pessina by Branconi’’^5^ by Anna Rosina de Gasc (Braunschweig, 1770) and the ‘‘Portrait of Duke Carl Wilhelm Ferdinand, Hereditary Prince of Braunschweig’’^6^ by Pompeo Batoni Cavallino (Rom, 1767).

**FIGURE 1:**
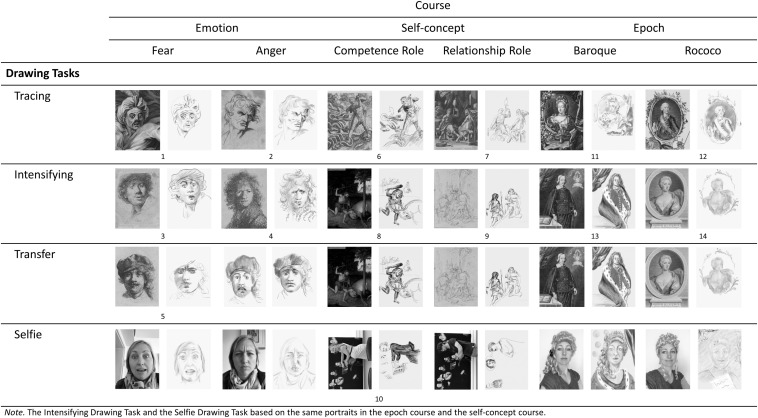
Overview over the pictures used in the visual-arts education program for drawing.

In the second course session, the adolescents engaged in portrait drawing by themselves. Here, they could develop their crafts and actively apply the knowledge obtained in the first session on the specific course content by working on various drawing assignments that where related to artworks in the museum. All artworks and assignments used are schematically presented in Figure 1 for all courses. The Appendix (Figure A1) contains the names of the portraits used for drawing. The assignments were presented in a sequence of increasing difficulty and growing demands for creative problem solving. Thus, they required students to engage and persevere to create something visible they had envisioned before.

For all drawing assignments a drawing app on a tablet computer was provided that was specifically designed for these assignments. The app comprised a selection of useful digital tools we considered helpful for supporting participants in engaging in their artistic projects, developing crafts, implementing the ideas they envision, exploring different design possibilities and expressing themselves. It contained the artworks for the drawing assignments as templates, allowed for taking selfies as templates for drawing, comprised simple editing functions (e.g., undoing or redoing strokes), enabled tracing of the templates with different intensities as well as zooming of templates in order to support the drawing of details. Additionally, the app allowed to record and replay the entire drawing process, which could be used at the end of the course session for students’ presentation and discussion of how their own drawings were developed.

In a first step, in the emotion course, artworks characterized by strong emotional facial expressions had first to be *reproduced* by students who were asked to focus on drawing the crucial characteristics in the artwork that revealed the emotions expressed. Students received two portraits with clearly recognizable emotions, namely fear and anger, and were asked to trace these portraits in the first step (Figure 1, *Tracing- Drawing-Task* 1, 2). In a second step, the emotionality of two portraits had to be *intensified* by means of drawing. Here, the adolescents received an ambiguous facial expression (e.g., a Rembrandt portrait whose facial expression might show surprise or fear). The students were asked to envision and draw this portrait in a way that it displays a clearly recognizable emotional expression of fear (Figure 1, *Intensifying-Drawing-Task* 3, 4). Finally, there was another task (Figure 1, *Transfer-Drawing-Task*, 5) in which the students were given a neutral painting that they should envision and draw in a way that it displays different emotions (anger, fear and as a transfer assignment the emotion sadness).

In the self-concept course, students were first provided with two paintings of the mythical figure Hercules showing the figure either in a “competence-oriented” role as a strong and invincible hero fighting against the Hydra or in a “relationship-oriented” role as a vulnerable lover dominated by Omphale (Figure 1, *Tracing- Drawing-Task* 6, 7). They were asked to trace these paintings with a focus on the characteristic features showing how different Hercules might perceive himself in these two conflicting roles. Subsequently, students again received two pictures showing Hercules either in a competence- or relationship-oriented role. However, here Hercules was less clearly recognizable as a hero or lover in these paintings. The task of the students was to envision and draw these pictures of Hercules in a way that he would be clearly recognizable as a hero in the competence-oriented role or as a lover in the relationship-oriented role (Figure 1, *Intensifying-Drawing-Task* 8, 9).

In the epoch course, students first received two characteristic portraits from the Baroque and Rococo periods and were also asked to trace them, focusing on the characteristic features of the particular epoch (Figure 1, *Tracing-Drawing-Task* 11, 12). Secondly, students received an ambiguous portrait of a scientist and a lady. The task of the students was to envision and draw the scientist as a baroque prince by using colors and characteristic elements, e.g., dark-colored heavy curtains, or the lady as a classical rococo lady (e.g., with flowers, pastel-colored clothes, etc.; Figure 1, *Intensifying-Drawing-Task* 13, 14).

In the third session of the course, the focus was again on drawing processes. This time, however, participants were stronger stimulated to express themselves by drawing portraits based on their own selfies. The selfies were created with the tablet computer and could be used as drawing templates. Depending on the content of the course, the selfies were used for staging an emotional situation, a role conflict or a period of art history. In the emotion course, the students were asked to take a selfie of themselves looking a little sad or a little angry and to create portrait drawings out of these selfies that display the fully developed expressions of the emotions “anger” and “fear.” In the self-concept course, we used the “Family Picture” of de Vos again (Figure 1, *Selfie-Drawing-Task*, 10). We printed a life-size version of the painting and cut out the central figure sitting at the piano. Students could place themselves behind the cut-out area to create a selfie of themselves as the pianist in the painting. They were asked to use the selfie as a template for a drawing in which they envisioned themselves in a competence-oriented role such as a pianist in a concert hall or in a music lesson. In a second relationship-oriented assignment, students should use the selfie to envision themselves in the situation that they were supposed to marry someone in the portrait they do not love. The focus of the drawing assignments was on envisioning how they would look and feel in these two situations. In the epoch course, the students had the opportunity to use wigs to stage themselves as persons of the Baroque or Rococo period. They used the selfies taken with these wigs as templates for envisioning and drawing themselves as historic personalities. The self-reference introduced in the third course session by staging one’s own person was assumed to deepen the processing of the courses’ contents (Symons and Johnson, 1997). As in the second course session, the drawing phase ended with a mutual presentation and reflection on participants’ own drawings. Following the third course session, the adolescents filled in the posttest.

### Stimuli and Apparatus

#### Animated Morph Task

**FIGURE 2:**
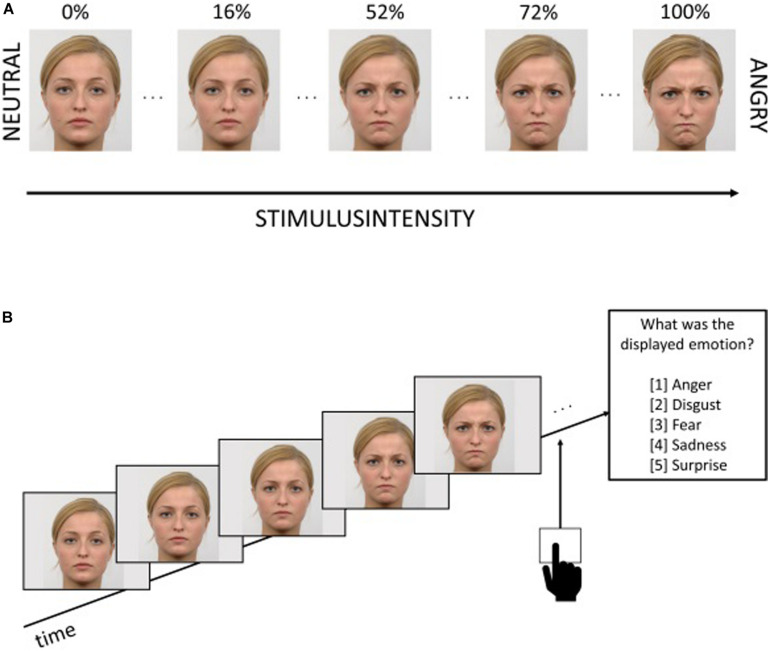
**(A)** Exampleof the stimuli used in the animated morph task. Stimuli were selected from the Radboud Face Database (Langener et al., 2010). **(B)** Trial procedure used in the animated morph task in which emotion intensity required until participants responded (i.e., participants sensitivity) and the accuracy of emotion recognition was measured (Schönenberg et al., 2014).

Digitized color photographs of five male and five female models displaying affective facial expressions of five basic emotions (anger, sadness, fear, surprise, and disgust) were selected from the Radboud Face Database (Langener et al., 2010). Differences in the stimulus material in color and luminance were adjusted by using Adobe Photoshop CS4. The stimulus intensity was systematically modified by blending neutral expressions (0%) into full emotional one’s (100%) in 51 unique levels (2% incremental steps, each step was presented for 250 ms) using a morphing procedure (FantaMorph Software, Abrosoft, Bejing, China). The stimulus material consisted of 50 trials (10 models × 5 emotions). Each stimulus was presented only once per test to minimize repetition effects. Students needed approximately 36 s on average to classify a stimulus into one of the five basic emotions (15 min for the whole task). For stimulus presentation, data collection and data decryption, we used the Presentation software version 20.0 (Neurobehavioral Systems, United States) on a tablet screen (12,9” iPad Pro, 2732 × 2048 Pixel 264 ppi, iOS 12.1). Figure 2A shows an example of the stimulus presented as a video sequence developing slowly from neutral to emotional facial expressions. Students were instructed to recognize the emotional facial expression as quickly and accurately as possible. When the students were able to identify the emotional facial expression, they had to press a stop button and the video presentation stopped immediately with the facial emotional expression disappearing. Subsequently, students had to indicate, which of the five emotions the face had expressed by means of a multiple-choice item (Figure 2B).

#### Self-Concept Task

**FIGURE 3:**
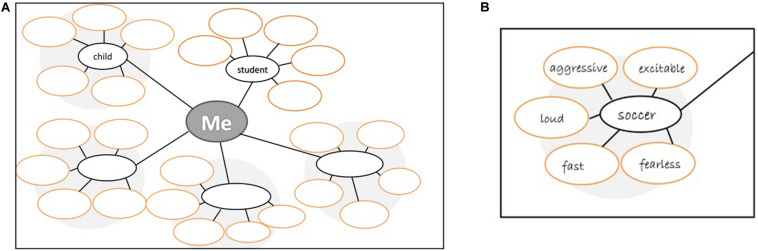
**(A)** Form used for the self-concept task. **(B)** An example for one self-aspect of a person (Kastner et al., 2020b).

Based on Linville’s (1985) self-complexity measure, we developed a graphical task in paper-pencil format to measure the complexity and structure of participants’ self-concept. In this task, students had to use a graphical form with five big circles to note down three important roles in their lives in addition to the two roles child and student that were already filled in two of the circles. Furthermore, they had to use adjectives to characterize themselves in each of the five roles by writing these adjectives into smaller circles surrounding the bigger circles containing the roles. Five adjectives could be filled into the five smaller circles surrounding each role. Students could either select adjectives from a prepared list of 100 adjectives (Aron et al., 1992) or generate adjectives by themselves. The prepared list of adjectives was designed to represent a wide range of characteristics that persons use to think about themselves and included both positive and negative characteristics. The number of roles and adjectives written down were used to calculate a measure of self-complexity. Figure 3 shows the task (A) and an example of an adolescent (B).

#### Control Variables

To test Hypothesis 3 related to individual differences between participants with regard to their general ability to cognitively and affectively empathize with others, we included the *Toronto Empathy Questionnaire* (TEQ, Spreng et al., 2009) in this study. The TEQ is a self-reporting questionnaire that provides a standard compound measure comprising different aspects of empathy. A high cumulative score in the TEQ indicates highly developed abilities to empathize with the thoughts and feelings of others. In addition to the TEQ, we obtained demographic data on participants preconditions with regard to age, media usage, art interest, art mark, visits to art exhibitions, drawing skills, drawing as a hobby, and photography as a hobby.

### Analytic Approach

Data analyses were performed using the lme4 package (Bates et al., 2012) in the free software R (R-Core-Team, 2018). For the animated morph task, we analyzed accuracy (i.e., percent correct) and sensitivity (i.e., emotion intensity required until participants responded) as two dependent variables. We used linear mixed-effect models with random intercepts for participants and random slopes for emotion for each participant to investigate the effects of course and emotion on the dependent variables. Within the five emotions presented in the task, we distinguished between drawn emotions (anger, fear) and not-drawn emotions (surprise, disgust, sadness) to examine how specific the effects of drawing are. As course effects might depend on participants who already developed empathic skills (Hypothesis 3), we included participants’ pretest TEQ score as well as their age and their initial emotion recognition performance in the pretest before participating in the course program as covariates in the models.

For the self-concept task, we also derived two dependent variables as indicators for self-complexity: the number of unique adjectives (without repetition) used for self-description and the number of self-generated adjectives, both divided by the number of roles elaborated on in the graphical form. Therefore, we calculated the average number of adjectives per role (APR, cf. Eq. 1), separately for self-generated adjectives only and for all adjectives used including those from Aron et al.’s (1992) list. The rationale for these measures was as follows: Depending on how many of the five roles were elaborated on in the graphical form, students could fill in a maximum of 25 adjectives (i.e., 5 adjectives per role). If identical adjectives were used in the description of several roles this would reduce the overall number of unique adjectives used (indicating lower levels of self-complexity). Additionally, if students would not just pick adjectives out of the preselected list of possible adjectives but felt that they would need more specific adjectives to describe a specific role adequately, this was counted as indicating higher levels of self-complexity. Since no within factors were included in the models for the self-concept task, we calculated a multiple regression model with the categorical predictors course and TEQ and age and pretest as metric covariates.

(1) $$A\,P\,R=\frac{\text{Number}\,\text{of}\,\text{adjectives}}{\text{Number}\,\text{of}\,\text{roles}}$$

## Results

Data and analysis scripts for all reported results can be found in Supplementary Material. For the analyses in this paper, 224 data sets with complete pre- and post-measurements were included for the animated morph task and 209 data sets could be used for the self-concept task. A table with number of participating students for each experimental condition and time point can be found in Supplementary Table S1.

### Demographic Data

To test whether the experimental groups differ with regard to their demographic data (age, media usage, art interest, art mark, visits to art exhibitions, drawing skills, drawing as a hobby, and photography as a hobby) and their pretest scores (for accuracy, sensitivity, APR and self-generated APR) separate analyses of variance were conducted. Table 1 shows the means and standard deviations for all variables. Results indicate no differences between the courses, except for age and the pretest scores in the animated morph task with regard to accuracy as well as to sensitivity. Students in the emotion course were younger on average than the ones in the other two courses, *F*(2, 244) = 10.85, *MSE* = 30.87, *p* < 0.001, partial η^2^ = 0.08, and had a lower emotion recognition performance in the pretest, *F* = 4.89, *MSE* = 0.25, *p* = 0.008, partial η^2^ = 0.01, while also being less sensitive in the pretest, *F* = 6.28, *MSE* = 924.23, *p* = 0.002, partial η^2^ = 0.01 (all other results for the demographic variables can be found in Supplementary Table S2). It has been shown that emotion recognition performance is associated with age (Tonk et al., 2007) and this can also be seen in our data. The correlation between age and the percentage of correctly recognized emotions in the pretest was significant, *r* = 0.13, *t*(1,033) = 4.34, *p* < 0.001, 95%CI = [0.07, 0.19]. For sensitivity, however, no significant correlation with age was found, *r* = –0.03, *t*(1,043) = –1.04, *p* = 0.297, 95%CI = [–0.09, 0.02]. Based on these results, age and pretest scores were used as covariates in all analyses.

**TABLE 1:** Means and standard deviations for sociodemographic variables and pretest scores for all dependent variables for each experimental condition.

	**Course**					
	
	**Epoch**		**Emotion**		**Self-concept**	
			
	***M***	***SD***	***M***	***SD***	***M***	***SD***
Age	15.15	1.80	14.27	1.68	15.64	1.41
Media usage	2.88	1.71	3.06	1.71	2.82	1.64
Art interest [1 very low, 2 low, 3 intermediates, 4 frequently, 5 always]	2.62	1.15	2.54	1.16	2.63	1.12
Art mark [1 very good, 2 good, 3 satisfactory, 4 sufficient, 5 poor]	2.21	0.93	2.33	1.13	2.04	0.79
Art exhibition [1 never, 2 1–3 times, 3 3–6 times, 4> 6 times]	1.87	0.80	1.88	0.59	1.86	0.72
Drawing skills [1 very good, 2 good, 3 satisfactory, 4 sufficient, 5 poor]	2.98	1.21	2.82	1.25	2.96	1.24
Drawing hobby [1 rarely or never, 2 sometimes, 3 frequently, 4 always]	1.88	0.97	1.99	1.05	1.73	0.84
Photography hobby [1 rarely or never, 2 sometimes, 3 frequently, 4 always]	2.40	0.91	2.30	0.97	2.32	0.97
Accuracy_Pretest_	76.25	22.90	71.72	23.94	77.75	21.35
Sensitivtiy_Pretest_	60.78	11.94	63.83	11.56	60.69	13.11
APR_Pretest_	3.38	1.11	3.61	0.93	3.48	1.15
APRself_Pretest_	0.76	0.67	0.82	0.72	0.72	0.55

### Animated Morph Task

#### Data Preparation for Analysis

**FIGURE 4:**
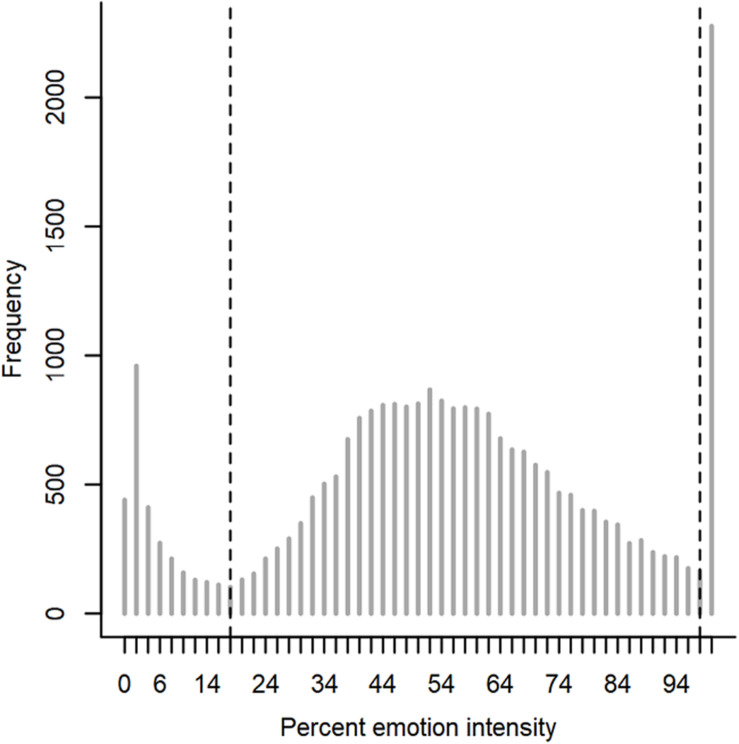
Frequency distribution of students’ overall response behavior as a function of emotion intensity (0–100%) with exclusion criteria: In the accuracy analysis only trials with an emotion intensity <18% were excluded from the analysis. In the sensitivity analysis trials with an emotion intensity <18 and >98% were excluded from data analysis.

As described in “Materials and Methods” section, we were interested in two variables: (1) the accuracy in terms of the percentage of correctly recognized emotional facial expression, and (2) the mean emotion intensity required by participants for the correctly recognized emotional facial expression (i.e., sensitivity). Five emotional facial expressions (angry, sad, fearful, disgusted, and surprised) of 10 individuals at two points in time (pre, post) had to be recognized in the animated morph task. We defined separate exclusion criteria for the *Accuracy* and the *Sensitivity Analysis*. For both analyses, we used a data driven approach for defining exclusion criteria based on the frequency distribution of reactions per emotion intensity (Figure 4). Generally, two behavioral patterns were interpreted to indicate that the instruction to recognize emotions as quickly and accurately as possible was not complied with: (1) Students waiting until the emotional expression was fully developed (100% emotion intensity) before providing a judgment, and (2) students trying to complete the task as quickly as possible without working on the task conscientiously thereby completely sacrificing accuracy. Figure 4 shows these two behavioral patterns as accumulations between 0 and 16% emotion intensity and at 100% emotion intensity.

For the accuracy analysis all trials in which participants responded without caring for accuracy (emotion intensity < 18%) were excluded. As a result, 11% of the trials were excluded from the analysis. These trials had an average accuracy at chance level for all courses (about 20% correctly solved trials with five possible answering alternatives, Supplementary Table S3). Trials in which participants waited up to 100% of emotional facial expression were not excluded in the accuracy analysis, although it can be assumed—based on the distribution of emotion intensities required—that the adolescents actually did recognize the emotion before it was fully developed. However, for the accuracy analysis it is most important to have the information whether or not an emotion was recognized correctly and that can be answered even when the exact intensity required for this decision is not known. At the aggregated levels of the percentage of correctly solved trials per emotion, only average values were included in the analysis that were based on at least five valid trials out of the 10 trials presented per emotion per test.

For the sensitivity analysis, only correct answers were included in the analysis. The exclusion criterion was slightly different than for accuracy. As before, trials in which participants responded without caring for accuracy (emotion intensity < 18%) were excluded. In contrast to the accuracy analysis, however, we also excluded those trials in the sensitivity analysis in which participants waited up to 100% of emotional facial expression before they reacted. Figure 4 shows this pattern as a massive accumulation at 100% emotion intensity. The rationale for this exclusion is again that it can be assumed—based on the distribution of emotion intensities required—that the adolescents actually did recognize the emotion before it was fully developed—but did not stop the morphing. Therefore, these data contain no information with regard to the sensitivity in these cases, which is, however, crucial for a sensitivity analysis.

In sum, the reason for excluding these 100% intensity trials in the sensitivity analysis but not in the accuracy analysis relates to the meaningfulness of the information obtained: On the one hand, we do know whether these trials were answered correctly or not and this information can be meaningfully used as an indicator for the performance distribution with regard to accuracy, but on the other hand, we do not have any information about the sensitivity required for emotion detection when the morphing was only stopped after the 100% emotion intensity was displayed before judgements were provided. The mean sensitivity for these excluded judgments was between 5.01 and 7.80% for the different courses (see Supplementary Table S3). Accordingly, 20% of the data points had to be excluded from the sensitivity analysis due to 100% values. One student had to be excluded from the data analyses entirely as he reacted in all trials when the emotional intensity of 100% was reached. For all other students, only individual trials were excluded.

#### Accuracy Analysis

**FIGURE 5:**
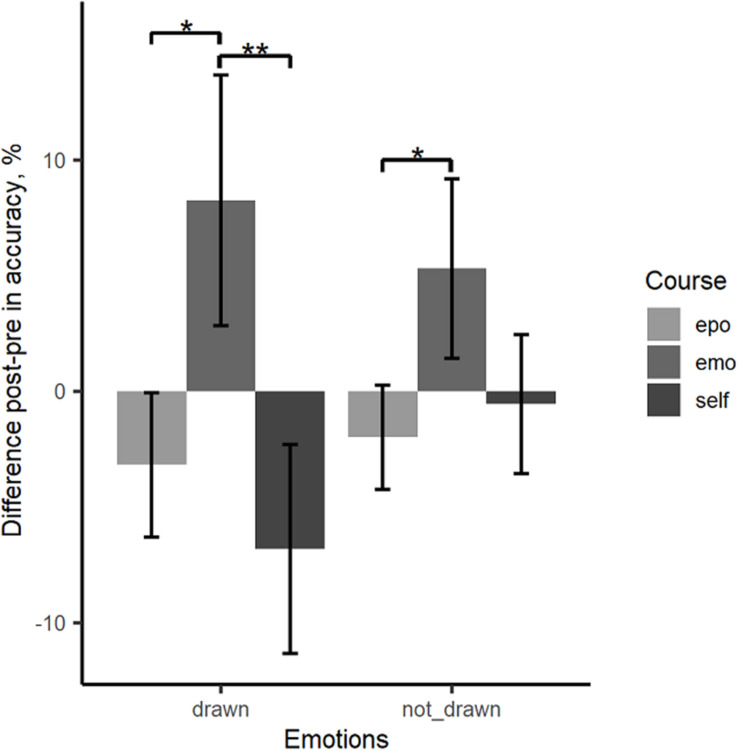
Mean change scores for the drawn emotions (anger, fear) and the not-drawn emotions (disgust, sadness, surprise). The mean change scores for accuracy are calculated from P(correct)_post_ – P(correct)_pre_; epo, epoch course; emo, emotion course; self, self-concept course. Error bars represent the standard error of the mean.

As described in the analytic approach, we used linear mixed-effects models to study specific course effects on emotion recognition performance. We included age and pretest score as covariates in the analyses. TEQ was considered as a discrete variable with high vs. low empathy (via median split). Type of emotion (drawn vs. not-drawn) was introduced as a fixed within participant factor. Change scores (y_post_—y_pre_) were used as dependent variable. Figure 5 shows change scores for the three courses separately for drawn (anger, fear) and not-drawn emotions (surprise, disgust, sadness). The accuracy performance differed depending on the course participated in. The emotion course was the only course where significant positive effects for the accuracy performance were found.

Table 2 shows the estimated parameters for the fixed and random effects of the model described above and their 95% confidence intervals for the change in accuracy from pre- to posttest as a dependent variable. Emotion drawn and the epoch course were defined as reference categories. The results show significant effects for emotion, course, age and pretest. There was no significant interaction between emotion and course. For the drawn emotions, *post hoc* tests showed significant differences between the emotion course and the epoch course, *t*(204) = 3.04, *p* = 0.017 as well as the self-concept course, *t*(208) = –3.62, *p* = 0.003. For the not-drawn emotion, only a significant difference between the emotion and the epoch course could be found, *t*(210) = 2.66, *p* = 0.050. The difference to the self-concept course was not significant, *t*(211) = –2.20, *p* = 0.158. There was no effect of high vs. low TEQ nor an interaction between TEQ and course. Overall, these results indicate specific effects of the emotion course on the emotion recognition performance as measured by recognition accuracy in the animated morph task.

**TABLE 2:** Estimated parameters for the change over time for accuracy.

**Fixed effects**				
	**Estimates**	**Std. error**	***t*-value**	**95%CI**

Intercept	9.86	7.44	1.32	[–4.84, 24.51]
**Emotion**	**7.18**	**1.91**	**3.76**	**[3.39, 10.94]**
**Course (emo vs. epo)**	**7.79**	**3.87**	**2.01**	**[0.17, 15.41]**
Course (self vs. epo)	–3.61	3.31	–1.09	[–10.12, 2.94]
TEQ	–3.23	2.07	–1.56	[–7.43, 0.89]
**Age**	**0.99**	**0.46**	**2.16**	**[0.09, 1.89]**
**Pretest**	**–38.84**	**3.07**	**–12.67**	**[–44.95, –32.74]**
Emotion × Course (emo)	–3.19	3.33	–0.96	[–9.76, 3.38]
Emotion × Course (self)	3.83	3.28	1.17	[–2.62, 10.30]
TEQ × Course (emo)	3.14	3.88	0.81	[–4.50, 10.83]
TEQ × Course (self)	–0.07	3.73	–0.02	[–7.45, 7.37]

**Random effects**				

	**Variance**	**Std. Dev.**	**Corr**	

Participant (Intercept)	92.93	9.64		
Participant (Emotion)	60.52	7.78	–0.82	
Residual	323.57	17.99		

*N = 196 adolescents. Emo, emotion course; epo, epoch course; self, self-concept course. Significant parameter estimates are marked bold.*

#### Sensitivity Analysis

**FIGURE 6:**
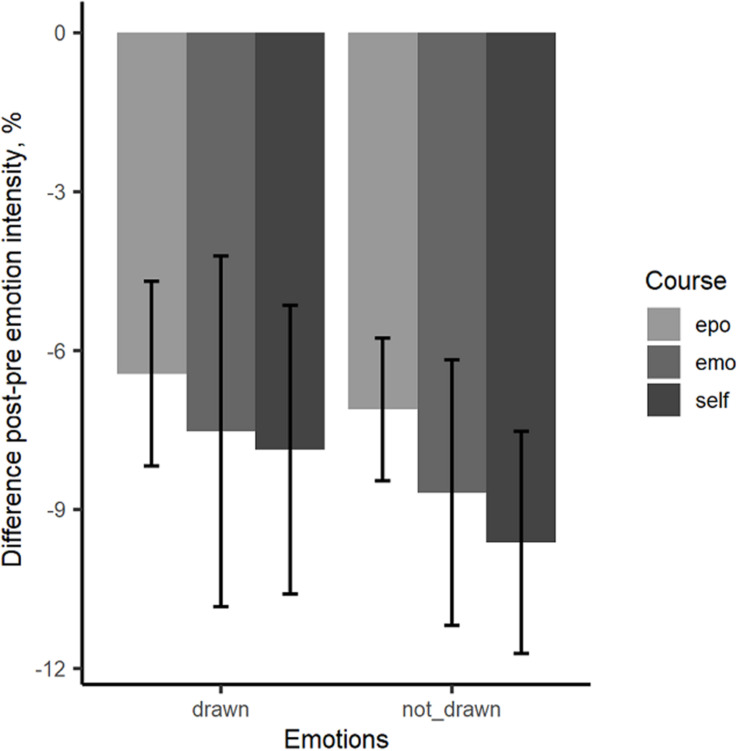
Mean change scores for the drawn (anger, fear) and the not-drawn emotions (disgust, sadness, surprise). The mean change scores for sensitivity are calculated from Sensitivity_post_ – Sensitivity_pre_; epo, epoch course; emo, emotion course; self, self-concept course. Error bars represent the standard error of the mean.

For the sensitivity analysis linear mixed-effects models were conducted as for the accuracy analysis. Results show significant effects for emotion and pretest (cf. Table 3). Both, the emotion course and the self-concept course, do not differ significantly from the epoch course. This pattern was confirmed by *post hoc* tests which showed no significant differences between the courses for drawn and not-drawn emotions. Only within the courses significant differences could be found between drawn and not-drawn emotions (*t*(200) = –4.03, *p* = 0.001 for the epoch course, *t*(201) = –3.89, *p* = 0.001 for the emotion course, and *t*(195) = –3.80, *p* = 0.001 for the self-concept course) which reflects the significant effect for emotion. All students were more sensitive after participating in one of the three courses. Subjects which had a low pretest score had a higher change from pre- to posttest. There was no effect of high vs. low TEQ nor an interaction between TEQ and course. Figure 6 shows the change scores for emotion intensity students need for correct emotion recognition for the three courses separately for drawn (anger, fear) and not-drawn emotions (surprise, disgust, sadness).

**TABLE 3:** Estimated parameters for change over time for sensitivity.

**Fixed effects**				
	**Estimates**	**Std. error**	***t*-value**	**95%CI**

**Intercept**	**31.72**	**6.22**	**5.10**	**[19.51, 44.05]**
**Emotion**	**–3.12**	**0.77**	**–4.04**	**[–4.64, –1.60]**
Course (emo vs. epo)	1.51	2.72	0.56	[–3.84, 6.88]
Course (self vs. epo)	–2.05	2.30	–0.89	[–6.58, 2.49]
TEQ	0.01	1.72	0.01	[–3.39, 3.42]
Age	0.31	0.38	0.83	[–0.43, 1.05]
**Pretest**	–**0.68**	**0.03**	**–20.81**	**[–0.74, –0.61]**
Emotion × Course (emo)	–1.29	1.36	–0.95	[–3.95, 1.38]
Emotion × Course (self)	–1.20	1.36	0.88	[–3.88, 1.48]
TEQ × Course (emo)	0.76	3.15	0.24	[–5.46, 6.97]
TEQ × Course (self)	1.35	3.12	0.43	[–4.79, 7.49]

**Random effects**				

	**Variance**	**Std. Dev.**	**Corr**	

Participant (Intercept)	72.80	8.53		
Participant (Emotion)	0.75	0.87	–1.00	
Residual	67.29	8.20		

*N = 206 adolescents. Emo, emotion course; epo, epoch course; self, self-concept course. Significant parameter estimates are marked bold.*

### Self-Concept Task

In the next step we analyzed whether specific effects of the self-concept course could be found with regard to the self-concept task used to assess the self-complexity of participants. To measure adolescents’ self-complexity, we calculated two variables: the number of adjectives per role (APR and the number of self-generated adjectives per role (self-generated APR). Self-generated adjectives refer to adjectives that were not on the list with adjectives provided for students (see “Materials and Methods” section). Change scores (y_post_—y_pre_) were used as dependent variables. Two participants were excluded from the analysis because they displayed a behavior on the task that indicated a complete lack of motivation and compliance, such as filling in the adjective “normal” in all five circles for all five roles in the graphical form. As for the animated morph task, we included age and pretest score as covariates in the analyses. Course and TEQ were introduced as discrete variables as before. For the self-concept task there were no random factors involved, therefore multiple regression models were conducted on the dependent variables.

**FIGURE 7:**
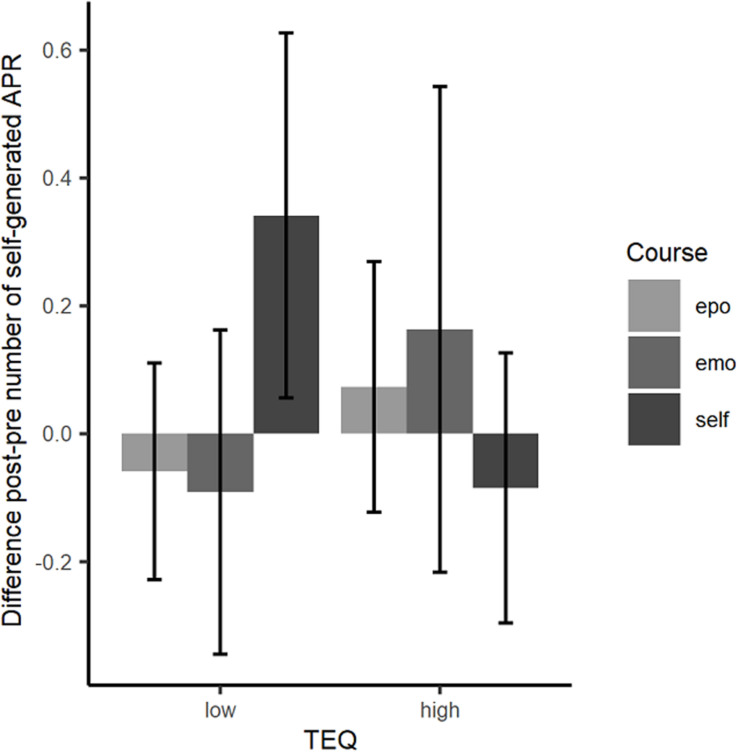
Mean change scores for the number of self-generated adjectives per role (APR). The mean change scores for APR are calculated from self-generated APR_post_ – APR_pre_, epo, epoch course; emo, emotion course; self, self-concept course, high and low values on the Toronto Empathy Questionnaire (TEQ). Error bars represent the standard error of the mean.

The results show that there were no effects of course, TEQ or age on the change scores for the number of adjectives per role (APR). Results are therefore not reported here but can be seen in Supplementary Table S4 and reproduced with the analyses scripts in Supplementary Material. Results for the self-generated APR showed a significant main effect of the course and a significant interaction between course and a significant interaction between course and TEQ, indicating that participants benefited most from the self-concept course and that this was particularly the case for low TEQ students (cf. Table 4). Moreover, we found a significant main effect for the number of self-generated adjectives in the pretest (cf. Table 4): Students with low pretest scores had a higher change score from pre- to posttest for self-generated APR. Figure 7 displays the change scores for the number of self-generated adjectives per role (APR).

**TABLE 4:** Estimated parameters for change over time for self-generated APR.

	**Estimates**	**Std. error**	***t*-value**	***p*-value**
**Intercept**	**0.83**	**0.38**	**2.21**	**0.028**
Course (emo vs. epo)	0.01	0.13	0.05	0.964
**Course (self vs. epo)**	**0.32**	**0.15**	**2.13**	**0.034**
TEQ	0.20	0.11	1.79	0.076
Age	–0.04	0.02	–1.50	0.137
**Pretest**	**–0.49**	**0.06**	**–7.72**	**<0.001**
TEQ (high) × Course (emo)	0.04	0.21	0.21	0.834
**TEQ (high) × Course (self)**	**–0.46**	**0.20**	**–2.26**	**0.027**

Residual standard error	0.56			
*R*^2^	0.28			

*N = 198 adolescents. Emo, emotion course; epo, epoch course; self, self-concept course. Significant parameter estimates are marked bold.*

### Test of Hypotheses

In this study we examined three hypotheses: (1) In the *Specificity Hypothesis (Hypothesis 1)*, we assumed that the emotion course should lead to specific improvements with regard to emotion recognition performance, whereas the self-concept course should lead to specific improvements with regard to self-complexity. (2) In the *Instructional Focus Hypothesis (Hypothesis 2)*, we assumed that the epoch course, which was used as a control group for the emotion and self-concept course, respectively, should neither yield positive effects on emotion recognition performance nor on self-complexity due to its lack of instructional focus on these constructs. (3) In the *Entry-level Hypothesis (Hypothesis 3)*, we assumed that particularly participants with lower entry levels of empathic skills prior to participating in the course might benefit more from courses addressing socio-emotional skills such as emotion recognition or self-complexity as compared to adolescents with already better-developed socio-emotional skills.

*Hypotheses 1* and *2* could be basically confirmed in our analyses. For the emotion course, course specific effects could be found for emotion recognition accuracy, whereas an unspecific positive effect on sensitivity could be found for all courses. For the self-concept task course-specific effects could also be found for the self-generated adjectives used to describe important roles taken by students. In line with *Hypothesis 3*, we also found that the TEQ had as moderating role for the latter variable. Persons with lower TEQ values self-generated more adjectives per role after than before the course, which was not the case for persons with higher empathic skills. This moderating effect of empathy was, however, only found for the self-concept course. We did not find any influence of prior empathy on emotion recognition performance in the animated morph task. Therefore, *Hypothesis 3* could be partially confirmed for the self-complexity but not for emotion recognition.

## Discussion

Previous research on (socio-emotional) transfer effects of visual-arts education programs can be characterized by three methodological shortcomings: There is a lack of (1) experimental comparisons, of (2) theory-based program designs, and of (3) objective measurement methods for outcomes. The aim of the present study was therefore to design and experimentally investigate theory-based visual-arts education programs from which specific and objectively measurable transfer effects are be expected due to a combination of *generic characteristics* of visual-arts engagement (e.g., a strong involvement with studio habits of minds) and *specific characteristics* of the programs with regard to methods used and contents addresses (e.g., empathy or self-complexity). We aimed at answering the following central research questions: (1) Are specifically designed visual-arts courses suitable for promoting socio-emotional transfer effects? (2) Are transfer effects course-specific or general in nature? (3) What role do personal preconditions with respect to socio-emotional skills play for course effectiveness?

### Specific Effects of the Visual-Arts Courses on Empathy and Self-Concept Development

With regard to the research question of whether specifically designed visual-arts education programs can promote socio-emotional transfer effects, we found encouraging results for both the emotion course and the self-concept course. For both courses beneficial outcomes could be demonstrated that were not found for the epoch course used as experimental control. In accordance with the *Specificity Hypothesis*, results showed significant improvements in the percentage of correctly recognized emotional facial expressions (accuracy) only in the emotion course. Surprisingly, however, this specific effect was limited to the accuracy measure but was not found for the sensitivity measure, which improved in all three courses from pre- to posttest. Various explanations for this generally improved sensitivity in all three courses are possible. First, a *familiarity effect* may have occurred, since participants already had seen the depicted facial emotional expressions in the pretest (Zajonc, 1968; Bornstein and D’Agostino, 1992). Although each emotional facial expression was presented only once per test, this single exposure might have been sufficient for students to enable them recognizing an emotion faster on the second encounter. Clinical studies using this animated morph task (Schönenberg et al., 2014) have also demonstrated that it takes only a small number of repetitions to yield this type of *familiarity effects.* Second, a *general learning effect* with regard to analyzing facial expression might have occurred due to the study of visual arts. For instance, Hetland et al. (2013) argue that the ability to observe precisely is trained through the studio habits of mind practiced during an engagement with visual arts. Better observation may have led to faster responses in the animated morph task. A third possible explanation might be given by assuming a decay in motivation leading to a faster response for all students in the posttest as compared to the pretest. Since the posttest was administered at the very end of the third course session, it might be possible that the adolescents were exhausted and wanted to finish the tasks as quickly as possible, thereby changing their criterion for speed-accuracy trade-offs. This might also explain why student’s accuracy in the epoch course and the self-concept course dropped from pre- to post-test despite an increased familiarity with the stimuli. It has to be noted however, that in the emotion course the increase in sensitivity was accompanied by an increase in accuracy, which seems to contradict exhaustion and motivational problems as potential causes of faster responses in the posttest.

Looking at the beneficial accuracy (and sensitivity) effects on emotion recognition performance within the emotion course more closely, it is interesting to note that the improvements in accuracy were found for both, drawn emotions (anger, fear) and not-drawn emotions (surprise, sadness, disgust), regardless of their individual difficulty (Montagne et al., 2007). This finding is consistent with studies in clinical psychology showing that an implicit (Schönenberg et al., 2014) or explicit (Dadds et al., 2006, 2012; Combs et al., 2008) direction of attention on relevant facial features might lead to improved emotion recognition performance. This suggests that the emotion course itself might have focused participants’ attention on facial features relevant for emotion recognition, thereby leading to an improvement in accuracy even for emotions that are not elaborated in detail in terms of being subject to the drawing assignments (although descriptively the accuracy changes seem to be more pronounced for the drawn emotions).

With regard to the second target construct investigated (self-concept development in terms of self-complexity), we were interested in whether a specific visual-arts course might be helpful to perceive one’s own person more differentiated in diverse social roles. As for the emotion course, a specific effect of the self-concept course on self-complexity was found. This effect was moderated by the individual’s general empathic skills that were obtained by means of a subjective questionnaire measure in the pretest (TEQ). As expected in the *Entry-level Hypothesis*, we found that adolescents with lower empathic skills seemed to benefit more from participation in the self-concept course than those with higher empathy scores. In particular, participants in the self-concept course with lower initial empathy scores generated more adjectives per role after course participation as compared to the pretest, which was not the case for students in the other courses. It has to be noted, however, that the newly developed measurement instrument for self-complexity might have underestimated the effect of the intervention due to ceiling effects: In the task used to measure self-complexity, we had already provided adolescents with two roles (student and child) and left only three roles for open response. Many of the adolescents had therefore already filled in the maximum number of roles and adjectives during the pretest, which is why these participants could not achieve any observable increases in the posttest.

Since we focused on self-concept development (in the sense of self-complexity) and empathy (in the sense of emotion recognition) as socio-emotional target constructs for transfer effects, we designed two “psychological” visual-arts programs, that we compared with each other but also with a control course focusing on periods of art history. Beyond the specific transfer effect of each of the two interventions, we found—in line with the *Instructional Focus Hypothesis*—that there were no specific effects of the epoch course on socio-emotional skills, such as emotion recognition or self-complexity. Thus, in sum, our three research questions can be answered as follows: (1) Specifically instructed art courses are suited (and required) for the promotion of socio-emotional skills, such as empathy or self-concept development. (2) Transfer effects are “course-specific” and not generic in nature. Therefore, specific transfer effects can only be expected if a course is designed properly for the target construct in question. (3) Socio-emotional preconditions of participants, such as general empathy, might play a role as moderators for socio-emotional transfer effects and can influence the effectiveness of an intervention. These results are based on experimental randomization and objective measurements and can, therefore, claim to provide rather strong empirical evidence as compared to other studies on transfer effects of art education programs. Moreover, the results highlight the important role of specific psychological theories and models for a successful design of art education programs intended to yield socio-emotional transfer effects Since only a few studies exist that successfully demonstrate transfer effects of art education programs on socio-emotional skills in the literature to date, and since only a few of these address the issue with robust methodological approaches (Winner, 2019), we believe that an expansion of the scientific approach outlined in this paper might provide a promising avenue for the further development of the research field.

### Role of Studio Habits of Mind for Transfer Effects

An important issue might be raised with regard to the “mixture” of the art-related and psychology-related ingredients we designed into the two “psychological” course programs. On the one hand, these courses are based on psychological theories and models of the socio-emotional skills targeted and on approaches and methods from instructional psychology on how to teach, practice, and reflect a particular skill or competence in order to enhance it. Focusing on these aspects, one might suspect that these courses are basically psychological empathy and self-concept courses embedded in a visual arts paradigm, rather than visual arts courses *per se*. On the other hand, however, our courses are based on the on museum pedagogical course concepts deployed in traditional art courses at the HAUM and they were further strongly enriched by engaging students in practicing different studio habits of mind, which can be considered to be quite specific for an involvement with visual arts. We worked with authentic pieces of art in an exhibition context and required students to closely observe, envision and create expressive drawings, to persist in these activities over the course of several weeks, thereby developing their crafts and their understanding of artistic worlds. Moreover, our course program comprised three central elements described by Hetland et al. (2013): First, a “*Demonstration- Phase/Knowledge-Acquisition-Phase*” was conducted in which central course contents were conveyed to the adolescents. Subsequent to acquiring a basic knowledge in each course, adolescents were given the opportunity to apply their knowledge in a *“Students-at-Work-Phase”* in the context of artistic drawing assignments that increased in their demands for crafts and creativity. Lastly, finished drawings were presented and discussed together in a *“Reflection-Phase”* in each course. Therefore, we would consider the course programs as a genuine and interdisciplinary mixture of authentic visual-arts engagements and psychological elaborations on the course contents. Probably, typical arts teachers conducting these course programs might notice an atypical strong psychological component integrated in the programs, but nevertheless they might still consider them to be “real” arts courses due to the equally strong art-typical elements. Moreover, we obviously would not doubt the general value and importance of the traditional “arts for art’s sake” perspective of most visual arts classes. Rather, the present study is intended to provide an answer to the question of why in many studies on visual-arts programs no effects “beyond art’s sake” could be found, for instance in the socio-emotional domains covered in this study that are not genuinely attached to the studio habits of mind. Therefore, our study can be seen as a proof of concept that visual-arts programs can be tailored as authentic contexts that allow for achieving positive transfer effects even in the socio-emotional domain—when properly designed.

As one important caveat, however, it has to be noted that our study does not yet allow to draw strong conclusions as to which of the elements integrated in the overall course program were crucial for the positive effects obtained. First, students were always asked both, to study and to draw portraits. Therefore, we cannot distinguish from our current data how relevant these two components were individually for the outcomes obtained. In particular, we don’t know yet how important the creative aspects of the drawing tasks such as intensifying, and selfie-drawing were for course success. Second, we do not know for sure by now whether similar course effects could be found when students would engage in studying and drawing non-artistic portraits based on photos form magazines or yearbooks, for instance. In other words, we have not tested directly for the role of the authentic art context yet. However, we are convinced that studying and drawing photos from a magazine might not lead to the same improvements on socio-emotional skills as looking at and engaging with visual art, due to the uniqueness of the engagement with visual art beyond just looking at a photo. Third, we do not know how important the authentic art museum context might have been for the course effects. However, based on research on the perception of authentic objects in museums and exhibitions we can assume that the museum context might play and important role in the acquisition of knowledge. Schwan and Dutz (2020), for example, were able to show that museum visitors dealing with authentic objects develop more situational interest and give them a stronger impression of being in touch with the historical periods of the exhibition.

Therefore, future studies should focus more closely on the individual course elements and their influence on the development of socio-emotional skills due to visual-arts programs. In other words, elements such as the drawing assignments (including specific aspects such as the self-reference due to selfie-drawing), or the reflection phases should be studied in isolation regarding their overall contribution to socio-emotional learning. Similarly, the different studio habits of mind-elements comprised in the course programs might be validated individually with regard to their contribution to the overall effectiveness of the intervention.

### Implications for Clinical and Health Research

With regard to the scope of the current study it has to be noted that we recruited an average sample of healthy adolescents as participants. Thus, we do not know, yet, whether our interventions have the potential to be also helpful for students at risk with regard to their socio-emotional skills, which would require testing the intervention in clinical populations. However, we used clinical paradigms to measure effects on emotion recognition (Jusyte et al., 2017) and self-complexity (Linville, 1985, 1987; Woolfolk et al., 2004). This suggests that there might be interesting synergy effects between clinical research and experimental research on the effects of engaging in visual arts. It might well be that our course programs, with its specific transfer effects, is particularly well suited to be applied in important clinical areas. For instance, Schönenberg et al. (2014) could show that a subtle training (sensitivity to emotional expressions training, SEE training) that implicitly directed attention to important regions of emotional faces (with slowly fading out the intensity levels of the presented expressions over the course of training sessions) was suitable to improve perceptual insensitivities to facial affect found in a group of incarcerated violent offenders. As our emotion course also yielded strong improvements in emotion recognition performance, it would be interesting to test the effectiveness of the intervention in a similar way in clinical studies.

Moreover, the approach of art therapy also indicates that visual-arts programs might be helpful in clinical disorders associated with impaired emotion recognition (e.g., autism, Durrani, 2019) or with a rigid and non-adaptive self-concept, characterized by a low ability to take another person’s perspective concerning a situation (Semrud-Clikeman, 2007). Unsurprisingly, many health programs in general address the promotion of the two socio-emotional skills addressed by our interventions with the aim to produce significant and lasting changes in psychopathology (Spence, 2003). Therefore, we think that the research approach used in the present study could be extended to also explore causal effects of art therapy in greater detail. In addition, visual-arts education programs might not only be valuable to support and augment therapies for clinical conditions but also help in the prevention of clinical disorders.

## Data Availability Statement

The original contributions presented in the study are included in the article/Supplementary Material, further inquiries can be directed to the corresponding author/s.

## Ethics Statement

The studies involving human participants were reviewed and approved by the Kommission für Ethik in der psychologischen Forschung, Eberhard Karls Universität Tübingen. Written informed consent to participate in this study was provided by the participants’ legal guardian/next of kin.

## Author Contributions

PG developed the research question and the experimental paradigm. LK, AJ, and PG designed and planned the experiment. SF, SC-T, AJ, and PG were involved in planning and supervising the work. LK and SN collected the experimental data in Braunschweig. LK and NU designed the figures and tables and performed the statistical analyses. LK, NU, and PG wrote the manuscript. All authors provided critical feedback and helped in every stage of the research, analysis, and manuscript.

## Conflict of Interest

The authors declare that the research was conducted in the absence of any commercial or financial relationships that could be construed as a potential conflict of interest.

## Figures and Tables

**FIGURE A1:** Names of portraits used in this study for drawing (Figure 1).

^1^ Johann Elias Ridinger: Asia. Mezzotint, Braunschweig, Herzog Anton Ulrich-Museum, Germany.	^8^ Lucas Cranach the Elder: Hercules and Diana’s hind. Oil Painting, 1537, Braunschweig, Herzog Anton Ulrich-Museum, Germany.
^2^ G. Stein: Representation of different emotional states. Copperplate Engraving, Wolfenbüttel, Herzog August Bibliothek, Germany.	^9^ Lorenzo Pasinelli: Hercules and Omphale. Oil on canvas, 1649–1700. Braunschweig, Herzog Anton Ulrich-Museum, Gallery of Prints, Germany.
^3^ Rembrandt: Self-portrait with cap, big eyes and open mouth. Etching and drypoint, 1630, Braunschweig, Herzog Anton Ulrich-Museum, Germany.	^10^ Cornelis de Vos: Family picture. Oil Painting, 1618. Braunschweig, Herzog Anton Ulrich-Museum, Germany.
^4^ Rembrandt: Self-portrait with wrinkled forehead. Etching, 1630, Braunschweig, Herzog Anton Ulrich-Museum, Germany.	^11^ Elias Christoph Heiss: Wilhelmine Amalia, Duchess of Braunscheig-Lüneburg. Mezzotint, 1674–1725. Braunschweig, Herzog Anton Ulrich-Museum, Germany.
^5^ Rembrandt: Self-portrait with beret. Etching and hand colouring, 1630, Braunschweig, Herzog Anton Ulrich-Museum, Germany.	^12^ Johannes Esaias Nilson: Leopold Count of Daun. Etching, 1741–1788. Braunschweig, Herzog Anton Ulrich-Museum, Gallery of Prints, Germany.
^6^ Cornelis Cort: Heracles defeats the Hydra of Lerna. Copperplate Engraving, 1563, Braunschweig, Herzog Anton Ulrich-Museum, Germany.	^13^ Melchior Küsel: Joseph Furttenbach. Copperplate Engraving, 1651. Braunschweig, Herzog Anton Ulrich-Museum, Gallery of Prints, Germany.
^7^ Egidius Sadeler: Hercules spins at the Omphale. Copperplate Engraving, 1590–1629, Braunschweig, Herzog Anton Ulrich-Museum, Germany.	^14^ Georg Friedrich Schmidt: Adrienne Lecouvreur. Copperplate Engraving, 1737–1755. Braunschweig, Herzog Anton Ulrich-Museum, Gallery of Prints, Germany.

## Footnotes

1 https://kulturerbe.niedersachsen.de/objekt/isil_DE-MUS-026819_opal_herzanulm_kunshe_GG490/1/

2 https://kulturerbe.niedersachsen.de/objekt/isil_DE-MUS-026819_opal_herzanulm_kunshe_GG_206/1/

3 https://kulturerbe.niedersachsen.de/objekt/isil_DE-MUS-026819_opal_herzanulm_kunshe_GG528/1/LOG_0000/#isil_DE-MUS-026819_opal_herzanulm_kunshe_GG528

4 https://kulturerbe.niedersachsen.de/objekt/isil_DE-MUS-026819_opal_herzanulm_kunshe_GG524/1/

5 https://kulturerbe.niedersachsen.de/objekt/isil_DE-MUS-026819_opal_herzanulm_kunshe_GG630/1/LOG_0000/#isil_DE-MUS-026819_opal_herzanulm_kunshe_GG630

6 https://kulturerbe.niedersachsen.de/objekt/isil_DE-MUS-026819_opal_herzanulm_kunshe_GG676/1/LOG_0000/#isil_DE-MUS-026819_opal_herzanulm_kunshe_GG676
