# Supplementary material for: Designing Visual-Arts Education Programs for Transfer Effects: Development and Experimental Evaluation of (Digital) Drawing Courses in the Art Museum Designed to Promote Adolescents’ Socio-Emotional Skills
Source: Front Psychol. 2021 Jan 18;11:603984. doi: 10.3389/fpsyg.2020.603984 (PMC7848093; doi:10.3389/fpsyg.2020.603984)
Supplement: Supplementary file 1 [file Data_Sheet_1.ZIP › Supplements/Supplementary_Material.docx]

Supplementary Material

# Supplementary Tables

Table S1.

*Number of students for each experimental course and time point.*

| Course | | | | | |
| --- | --- | --- | --- | --- | --- |
| Dataset morphing | | | | | |
|  | Epoch | Emotion | Self-concept | NA | Total |
| Pretest | 129 | 63 | 60 | 11 | 263 |
| Posttest | 127 | 66 | 53 | 0 | 246 |
| Pre & posttest | 118 | 55 | 51 | 0 | 224 |
| At least one | 138 | 74 | 62 | 11 | 285 |
| Dataset self-concept | | | | | |
|  | Epoch | Emotion | Self-concept | NA | Total |
| Pretest | 123 | 55 | 58 | 5 | 241 |
| Posttest | 120 | 53 | 55 | 0 | 228 |
| Pre & posttest | 112 | 47 | 50 | 0 | 209 |
| At least one | 131 | 61 | 63 | 5 | 260 |

Table S2.

*Results for ANOVAs for demographic variables testing for differences between experimental conditions.*

|  | *MSE* | *F* | *df1* | *df2* | *p* | *η^2^* |
| --- | --- | --- | --- | --- | --- | --- |
| Age | 30.87 | 10.85 | 2 | 244 | < .001 | 0.082 |
| Media usage | 1.02 | 0.35 | 2 | 246 | .703 | 0.003 |
| Art interest | 0.17 | 0.13 | 2 | 246 | .878 | 0.001 |
| Art mark | 1.31 | 1.42 | 2 | 246 | .244 | 0.011 |
| Art exhibition | 0.01 | 0.02 | 2 | 246 | .983 | < 0.001 |
| Drawing skills | 0.61 | 0.41 | 2 | 246 | .666 | 0.003 |
| Drawing hobby | 0.98 | 1.05 | 2 | 246 | .351 | 0.008 |
| Photography hobby | 0.29 | 0.33 | 2 | 246 | .719 | 0.002 |

Table S3.

*Means and standard deviations for accuracy for excluded trials for the morphing task for each experimental condition.*

|  | Course | | | | | |
| --- | --- | --- | --- | --- | --- | --- |
|  | Epoch | | Emotion | | Self-concept | |
|  | *M* | *SD* | *M* | *SD* | *M* | *SD* |
| Accucary | 19.26 | 39.45 | 19.72 | 39.81 | 19.73 | 39.84 |
| Sensitivity | 5.01 | 4.81 | 5.80 | 4.98 | 7.80 | 5.08 |

Table S4.

*Estimated parameters for change over time for APR.*

|  | Estimates | Std. error | *t*-value | *p*-value |
| --- | --- | --- | --- | --- |
| **Intercept** | **2.18** | **0.63** | **3.46** | **< .001** |
| Course (emo vs. epo) | –0.22 | 0.21 | –1.07 | .288 |
| Course (self vs. epo) | 0.25 | 0.25 | 0.99 | .324 |
| TEQ | 0.00 | 0.19 | 0.00 | .999 |
| Age | –0.02 | 0.04 | –0.50 | .616 |
| **Pretest** | **–0.60** | **0.06** | **–9.34** | **< .001** |
| TEQ (high) × Course (emo) | 0.39 | 0.35 | 1.10 | .272 |
| TEQ (high) × Course (self) | –0.11 | 0.34 | –0.31 | .756 |
| Residual standard error | 0.94 |  |  |  |
| R^2^ | .32 |  |  |  |

*N* = 198 adolescents. Emo = emotion course, epo = epoch course, self = self-concept course. Significant parameter estimates are marked bold.
